# Inch-Sized Thin Metal Halide Perovskite Single-Crystal Wafers for Sensitive X-Ray Detection

**DOI:** 10.3389/fchem.2021.823868

**Published:** 2022-01-05

**Authors:** Anbo Feng, Shengdan Xie, Xiuwei Fu, Zhaolai Chen, Wei Zhu

**Affiliations:** ^1^ State Key Laboratory of Crystal Materials, Institute of Crystal Materials, Shandong University, Jinan, China; ^2^ Institute of Radiation Medicine, Shandong Academy of Medical Sciences, Shandong First Medical University, Jinan, China

**Keywords:** perovskite single crystals, X-ray detectors, single-crystal wafers, sensitivity, integration

## Abstract

Metal halide perovskite single crystals are a promising candidate for X-ray detection due to their large atomic number and high carrier mobility and lifetime. However, it is still challenging to grow large-area and thin single crystals directly onto substrates to meet real-world applications. In this work, millimeter-thick and inch-sized methylammonium lead tribromide (MAPbBr_3_) single-crystal wafers are grown directly on indium tin oxide (ITO) substrates through controlling the distance between solution surface and substrates. The single-crystal wafers are polished and treated with O_3_ to achieve smooth surface, lower trap density, and better electrical properties. X-ray detectors with a high sensitivity of 632 µC Gy_air_^−1^ cm^−2^ under –5 V and 525 µC Gy_air_^−1^ cm^−2^ under –1 V bias can be achieved. This work provides an effective way to fabricate substrate-integrated, large-area, and thickness-controlled perovskite single-crystal X-ray detectors, which is instructive for developing imaging application based on perovskite single crystals.

## Introduction

In recent years, metal halide perovskite (MHP) materials have been demonstrated as promising candidates for sensitive X-ray detections due to their superior properties (Li Z et al., 2021), such as large atomic number, high carrier mobility and lifetime, and low-defect density (Wei et al., 2017; Cheng et al., 2019; Chen et al., 2019). There are two kinds of perovskite X-ray detectors: direct and indirect detection modes (Zhou et al., 2020). Compared to indirect detection which converts X-ray to a light signal, the direct detection mode which converts X-ray to an electrical signal has larger spatial resolution and higher sensitivity (Basiricò et al., 2019). Because of the strong stopping power and superior spatial resolution in X-ray, α-Se dominates direct conversion X-ray imaging, such as computed tomography (CT). But α-Se detectors have low sensitivity and require high dose for imaging, which brings cancer risk to the patients (Kasap et al., 2000). In comparison, the sensitivity of perovskite X-ray detectors are several orders of magnitude larger than that of commercial α-Se detectors, especially for hard X-ray (Wang et al., 2020; Deumel et al., 2021; Liu et al., 2021a; Peng et al., 2021).

Current state-of-the-art perovskite X-ray detectors are mainly made of bulk single crystals, which possess better electrical properties and higher stability than their polycrystalline counterparts due to the absence of grain boundaries (Wei et al., 2017; Liu et al., 2018; Yao et al., 2020; Liu et al., 2021b). Perovskite single crystals can be grown by the low-cost solution-based method, which is compatible with Si-based application-specific integrated circuits for signal readout (Wei et al., 2017; Geng et al., 2020). In general, perovskite single crystals with millimeter-sized thickness are in principle enough to fully attenuate the incident hard X-ray that is predominantly applied in medical imaging areas (Song et al., 2020). However, perovskite single crystals show weak anisotropic growth, leading to large crystal thickness, which not only reduces the vertical carrier collection yield but can also cause undesired cross-talk between pixels (Zhuang et al., 2019). To avoid these drawbacks, high-working bias is required to strengthen the vertical carrier collection (Li et al., 2020). Nevertheless, the monovalent ions in perovskite materials are prone to migrate under bias, which can cause fluctuation of dark and signal currents (Shao et al., 2016; Pan et al., 2017). Recently, highly sensitive and self-powered perovskite X-ray detectors are achieved through finely controlling crystal thickness and optimizing the carrier transport properties, which represents an effective way to eliminate ion migration and cross-talk between pixels (Wu et al., 2021). Another challenge comes from the lack of an established method to grow thin single crystals with size comparable to bulk crystals (Kim et al., 2017). In this case, it is important to develop an effective method to grow inch-sized thin perovskite single crystals and integrate them with substrates to meet real-world applications.

In this work, we report sensitive X-ray detectors made of millimeter-thick and inch-sized MAPbBr_3_ single-crystal wafers. The MAPbBr_3_ single-crystal wafers are directly grown on indium tin oxide (ITO) substrates, and the thickness is controlled by the distance between solution surface and substrates. O_3_-UV exposure has been used for the post-procedure in order to passivate traps on the single-crystal surface (Wei et al., 2016; Yao et al., 2021). Eventually, X-ray detectors with a high sensitivity of 632 µC Gy_air_^−1^ cm^−2^ under −5 V and 525 µC Gy_air_^−1^ cm^−2^ under −1 V bias can be achieved. This work provides an effective way to fabricate substrate-integrated, large-area, and thickness-controlled perovskite single-crystal X-ray detectors, which is beneficial to meet real-world applications.

## Materials and Methods

**Chemicals and reagents**: Methylamine (CH_3_NH_2_) (40 wt% in water), hydrobromic acid (HBr) (40 wt% in water), and lead bromide were obtained from stannic iodide produced by Xi’an Polymer Light Technology. N, N-Dimethylformamide (DMF, 99%) was purchased from Aladdin Reagent Ltd. Fullerene (C_60_), and 2,9-dimethyl-4,7-diphenyl-1,10-phenathroline (BCP) and poly (3,4-ethylenedioxythiophene)/poly (styrenesulfonate) PEDOT: PSS were purchased from Xi’an Polymer Light Technology.

**Synthesis of Methylammonium Bromide (CH**_**3**_ **NH**_**3**_ **Br**): CH_3_NH_3_Br was synthesized on the basis of the previously reported method in which HBr and CH_3_NH_2_ were reacted with the molar ratio of 1:1.2. To be specific, 125 ml of methylamine (40% in ethanol) was first put in a 1,000-ml round bottom flask under an ice bath; subsequently, 125 ml hydrobromic acid (40 wt% in water) was added drop by drop; in case a severe reaction took place between them, the mixture was made to react under constant stirring for 6 h. The initial CH_3_NH_3_Br product was collected by evaporating the mixture using the rotary evaporator at 60°C. Then, the initial powder was washed by absolute ethyl alcohol three times and anhydrous ethyl ether three times. Finally, the white CH_3_NH_3_Br powder was obtained by recrystallization in anhydrous diethyl ether for half an hour and then dried in a vacuum oven at 60°C for 12 h.

**Growth of substrate-integrated MAPbBr**_**3**_ **thin single-crystal wafers**: Initially, a small MAPbBr_3_ crystal seed was grown by the inverse temperature crystallization (ITC) method. Subsequently, the crystal seed was put on PEDOT: PSS-covered ITO substrates and then immersed into a saturated solution to promote further growth of the crystal seed until the desired size was reached. The crystal thickness was controlled by the distance between the substrate and solution surface precisely.

**X-ray Detector Device Fabrication**: C_60_ (40 nm) and BCP (3 nm) were thermally evaporated at a rate of 0.2 Å/s to form the charge transport layer. Cu (80 nm) was thermally evaporated at a rate of 0.8 Å/s to form the electrode.

## Characterizations

Powder X-ray diffraction (PXRD) patterns were measured on a SmartLab SE High-Resolution Diffraction System with Cu Kα1 radiation (*λ* = 1.54186Å) in the range of 5–90°(2θ) with a single-crystal thin film of 2 × 1 × 0.02 mm^3^ in size. Scanning electron microscope (SEM): The surface and cross morphology images were taken from a field emission microscope (Phenom Pharos). Steady-state absorption: Absorption spectra were determined using a U3500 Hitachi UV/Vis Spectrophotometer with the self-made testing mold. Device I-V characteristics were collected by using a Keithley 2,400 analyzer. AM 1.5-G irradiation (100 mW/cm^2^) was produced by a xenon-lamp–based solar simulator. X-ray was generated by an Amptek Mini-X2 tube with an Ag target, whose characteristic Kα peak is 22 keV. The X-ray dose rate was calibrated by a Radcal Accu-Gold^+^ 10X6-180 ion chamber dosimeter.

## Results and Discussion

**FIGURE 1:**
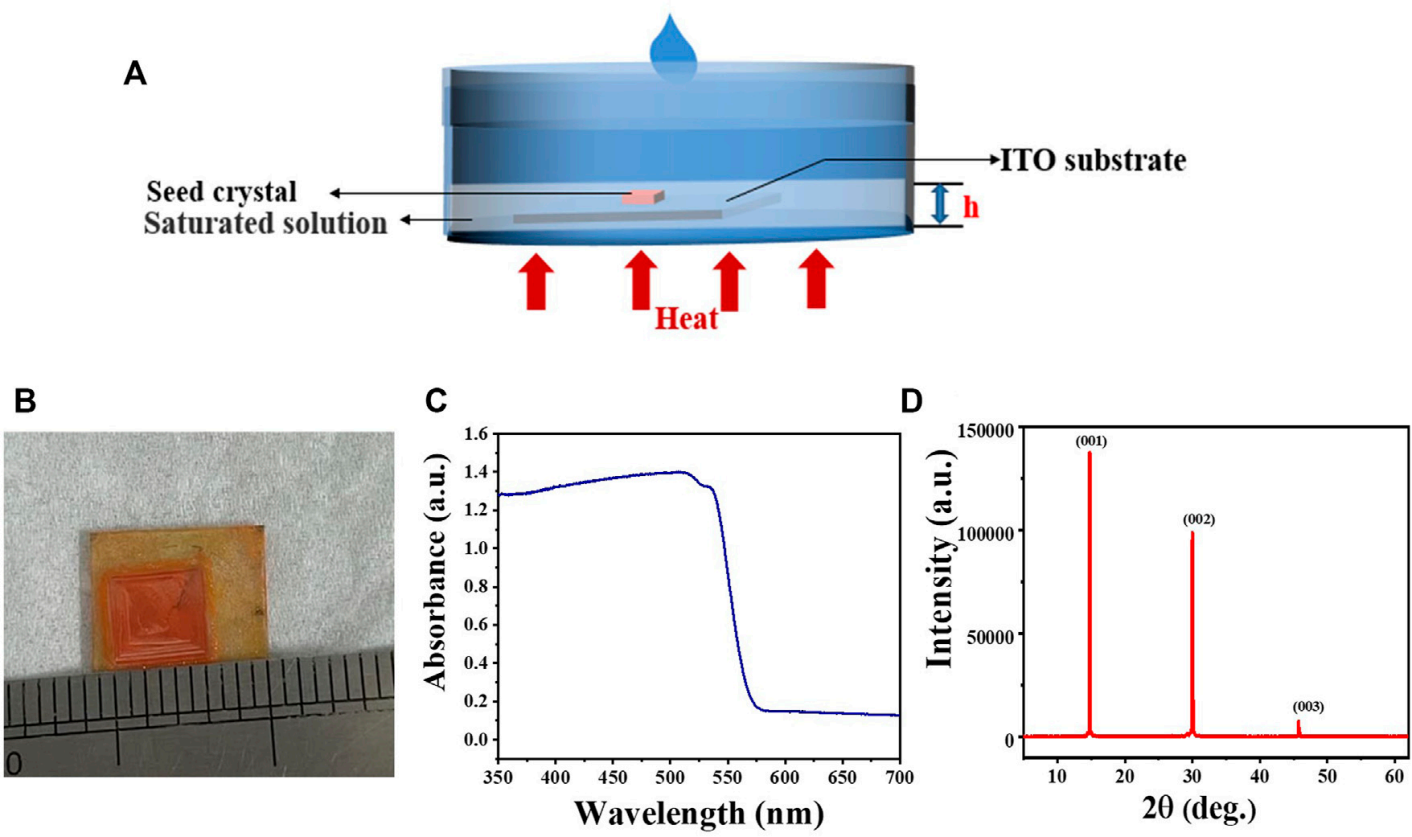
**(A)** Schematic illustration of the MAPbBr_3_ single-crystal wafer growth process. **(B)** Photograph of the as-grown single-crystal wafer attached to the ITO glass. **(C)** UV-Vis–NIR absorption spectra. **(D)** XRD pattern of the MAPbBr_3_ single-crystal wafer.

MAPbBr_3_ is a prototype perovskite material for X-ray detection application and therefore was selected for our investigation. To grow thickness-controlled perovskite single crystals, a space-confined strategy has been widely adopted (Liu et al., 2016; Chen et al., 2017; Alsalloum et al., 2021). But, the slow ion diffusion rate in the confined space can cause small crystal size and poor crystal quality (Cheng et al., 2019). Here, we try to control the crystal thickness by adjusting the distance between the solution surface and substrate, as illustrated in Figure 1A. Initially, a small MAPbBr_3_ crystal seed was obtained by the ITC method in which solubility decreases with increasing temperature. Subsequently, the crystal seed was put on PEDOT: PSS-covered ITO substrates and then immersed into a saturated solution to promote further growth of the crystal seed. To obtain high-quality single crystals, the temperature was increased slowly with a rate of 1°C/h to ensure the solution concentration was located in the metastable region. The vertical growth will stop when the single crystal is close to the solution surface while the lateral growth is not limited, thus resulting in thickness-controlled and inch-sized single-crystal wafers.

As shown in Figure 1B, the as-grown MAPbBr_3_ single-crystal wafers are square and semitransparent, indicating their high crystal quality. The lateral size of the single-crystal wafer is nearly 1 cm, and the thickness can be controlled at millimeter scale. The basic properties of the single-crystal wafers are characterized by UV-vis absorption and X-ray diffraction (XRD). As shown in Figure 1C, the absorption onset is 575 nm, which is consistent with previously reported values (Zhang et al., 2020). The XRD patterns show sharp diffraction peaks at 14.92°, 30.07°, and 45.77°, which can be assigned to (001), (002), and (003) planes (Figure 1D), suggesting that the exposed face of the single-crystal wafers is the (001) crystal plane. These results clearly show the successful growth of thin MAPbBr_3_ single-crystal wafers.

**FIGURE 2:**
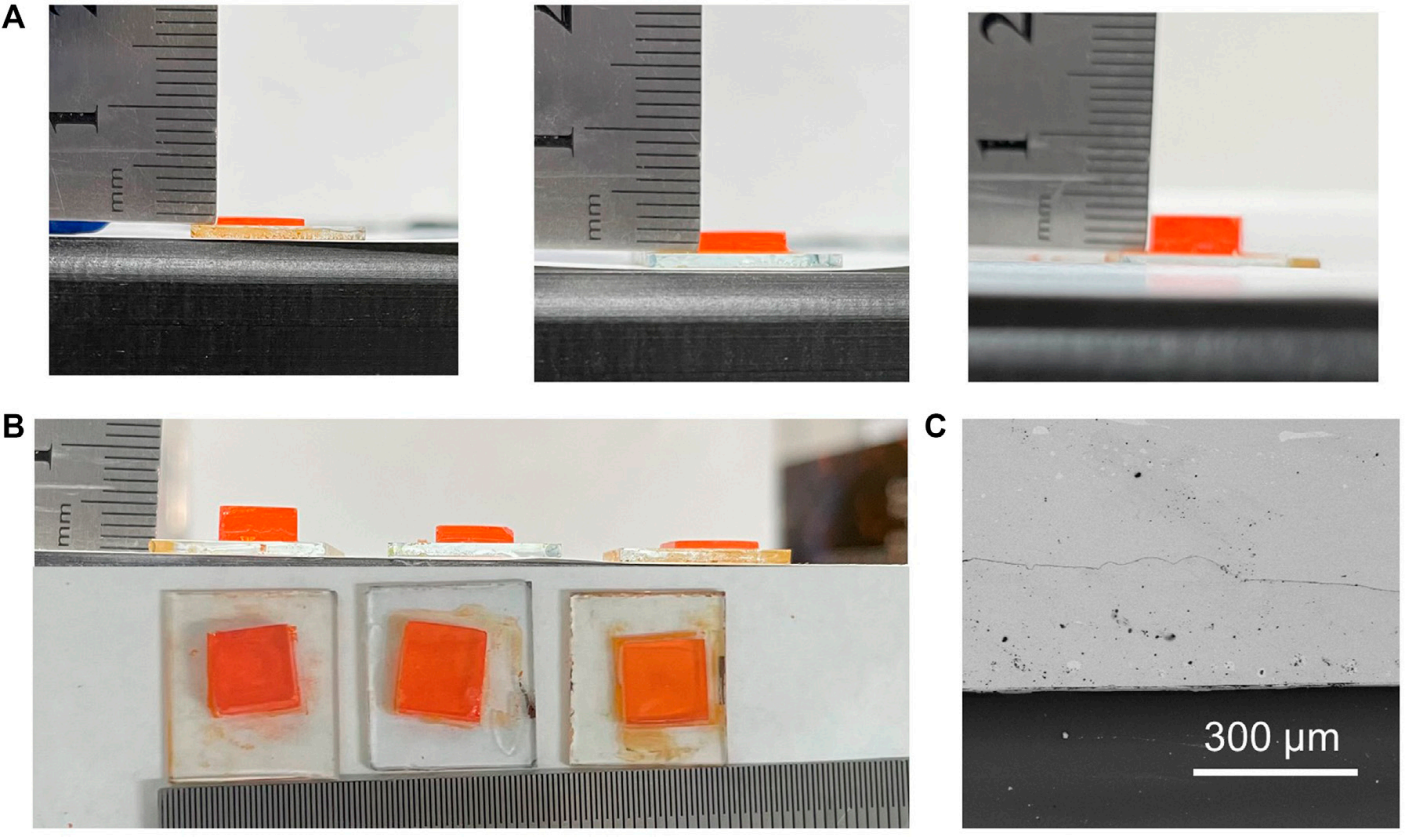
**(A)** Photographs of the as-grown MAPbBr_3_ single crystals with thicknesses differing from 1 to 3.5 mm. **(B)** Different thicknesses of the single crystal with similar size. **(C)** Cross-sectional SEM images of the MAPbBr_3_ single-crystal wafer.

X-ray with different photon energy levels has different penetration depths and requires different crystal thicknesses (Wei et al., 2016). To adjust the thickness of the single-crystal wafers, the distance between the substrate and solution surface is tuned. Figure 2A shows the photographs of MAPbBr_3_ single-crystal wafers with different thickness, which have similar shape and lateral size (Figure 2B). The crystal thickness can be controlled from ∼1 to ∼3.5 mm, satisfying full attenuation of soft and hard X-ray, respectively (Wei et al., 2016). Intimate interface contact is necessary for effective interface carrier injection and high X-ray response. To investigate the interface contact between the MAPbBr_3_ single-crystal wafers and ITO substrates, cross-sectional SEM measurement is conducted. As shown in Figure 2C, no voids and rain boundaries are observed between the MAPbBr_3_ single-crystal wafers and ITO substrates, confirming an intimate contact and single-crystalline character.

**FIGURE 3:**
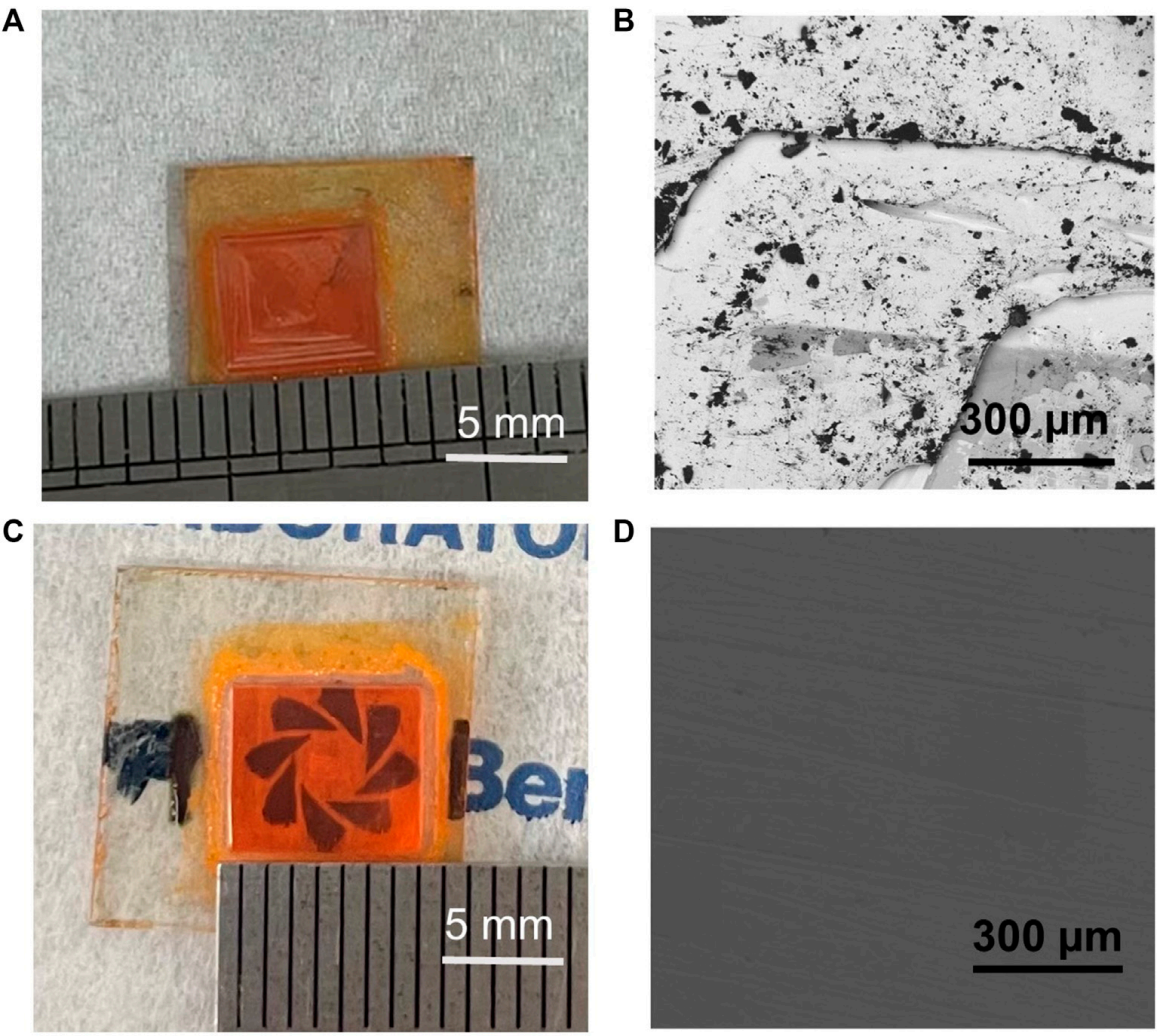
**(A)–(B)** Photograph and top view SEM of the as-grown single crystal. **(C)–(D)** Photograph and top view SEM of the polished single crystal.

From the photograph and top-view SEM image (Figures 3A,B), we find that a deep valley is observed on the surface of the as-grown MAPbBr_3_ single-crystal wafers, which will make it challenging for the continuous electrode. To overcome this problem, the crystal surface is polished by using 10,000-mesh sandpaper. It can be found that the crystal surface becomes relatively smooth after the polishing process, and the single-crystal wafers are still semitransparent (Figures 3C,D). The smooth surface can ensure deposition of the continuous electrode, which is important for electrical characterization and device fabrication.

**FIGURE 4:**
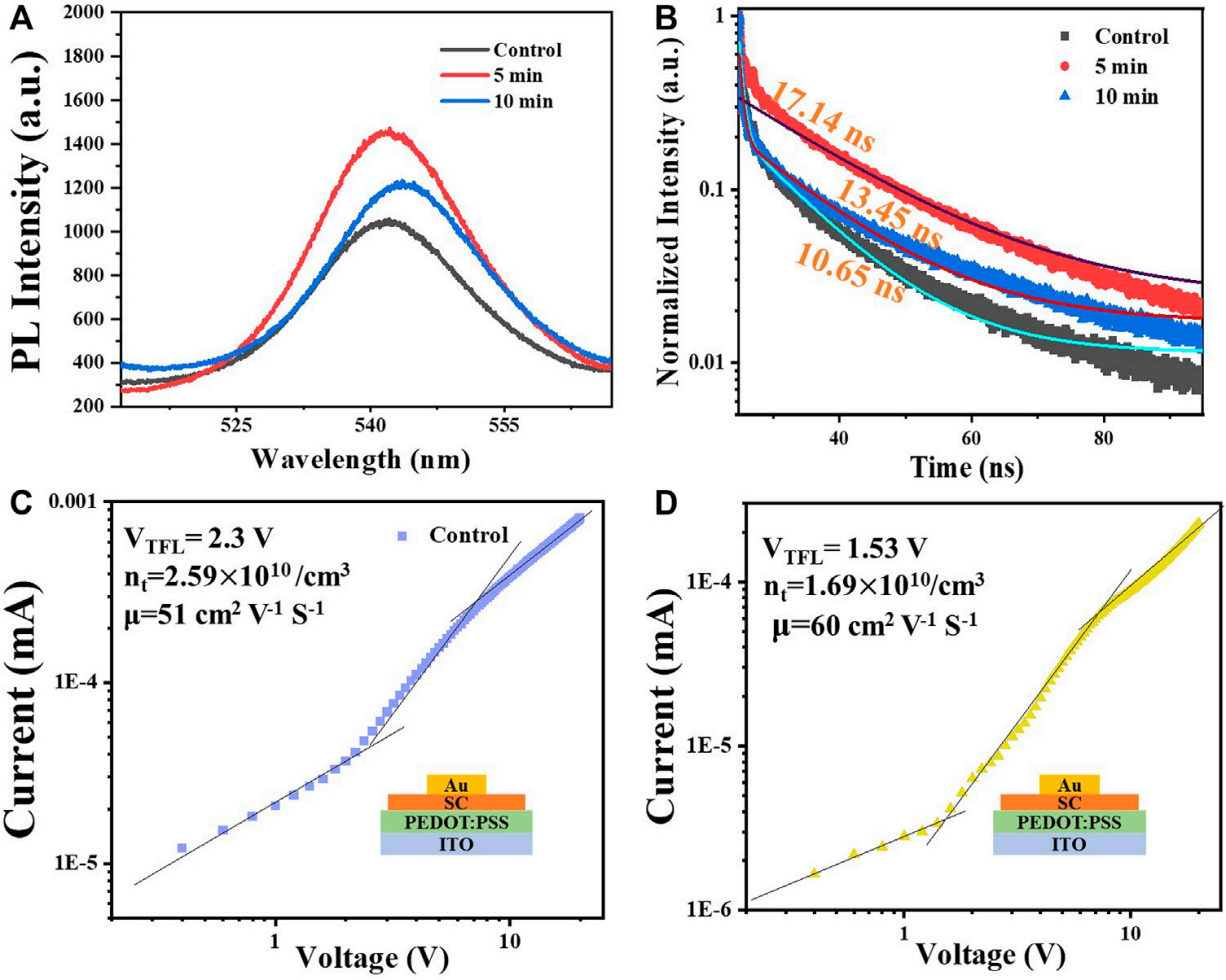
**(A)** Photoluminescence spectra and **(B)** time-resolved photoluminescence of the control sample and sample after O_3_–UV exposure for 5 and 10 min. **(C)–(D)** Space charge limited current (SCLC) measurement for the control and 5 min O_3_–UV-treated sample, and the structures of hole-only devices are displayed inset.

Low-defect density and superior carrier transport properties are important for high sensitivity of the detector devices. The surface trap passivation of the polished MAPbBr_3_ single-crystal wafers is conducted by O_3_–UV treatment. Photoluminescence was first conducted to preliminarily assess the trap passivation, as shown in Figure 4A; the PL intensity of the single-crystal wafer after 5 min of O_3_–UV exposure is stronger than that of the untreated sample, which can be attributed to the decrease of uncoordinated Pb atoms on the crystal surface; O_3_–UV exactly acts the role of the trap passivation during this process. Nevertheless, long-time exposure of O_3_–UV may bring about severe ion migration that is why PL intensity decreases after 10 min of O_3_–UV exposure (Li W et al., 2021). To obtain further analysis, the hole carrier mobility and carrier lifetime were measured by the transient time-resolved photoluminescence method (Figure 4B **)** and space charge limited current (SCLC) (Figures 4C,D). The hole-only devices with the structure of ITO/PEDOT: PSS/single crystals/Au were fabricated (Figures 4C,D inset**)**. The carrier mobility and trap density are calculated based on Equation 1 and Equation 2, respectively (Kim et al., 2020):

$$\;J_{\text{D}}=\frac{9\varepsilon \varepsilon_{0}\mu V_{\text{b}}^{2}}{8L^{3}},$$ (1)

where *J*_D_ is the dark current density, *ε* is the dielectric constant of perovskite single crystals, *ε*_0_ is the dielectric constant of vacuum, *μ* is the mobility, *V*_b_ is the bias, and *L* is the crystal thickness (Zhang et al., 2018).

$$V_{\text{TFL}}=\frac{en_{\text{t}}L^{2}}{2\varepsilon \varepsilon_{0}},$$ (2)

where *V*_TFL_ is the voltage at which all the traps are filled, *e* is the elementary charge, and *n*_t_ is the hole trap density. Without O_3_ treatment, the hole mobility and trap density are calculated to be 51 cm^2^ V^−1^ s^−1^ and 2.59 × 10^10^ cm^−3^, respectively. The O_3_ treatment increases the hole mobility to 60 cm^2^ V^−1^ s^−1^ and reduces the trap density to 1.69×10^10^ cm^−3^. According to the time-resolved photoluminescence measurement, the carrier lifetime increases from 10 to 17 ns after O_3_ treatment.

**FIGURE 5:**
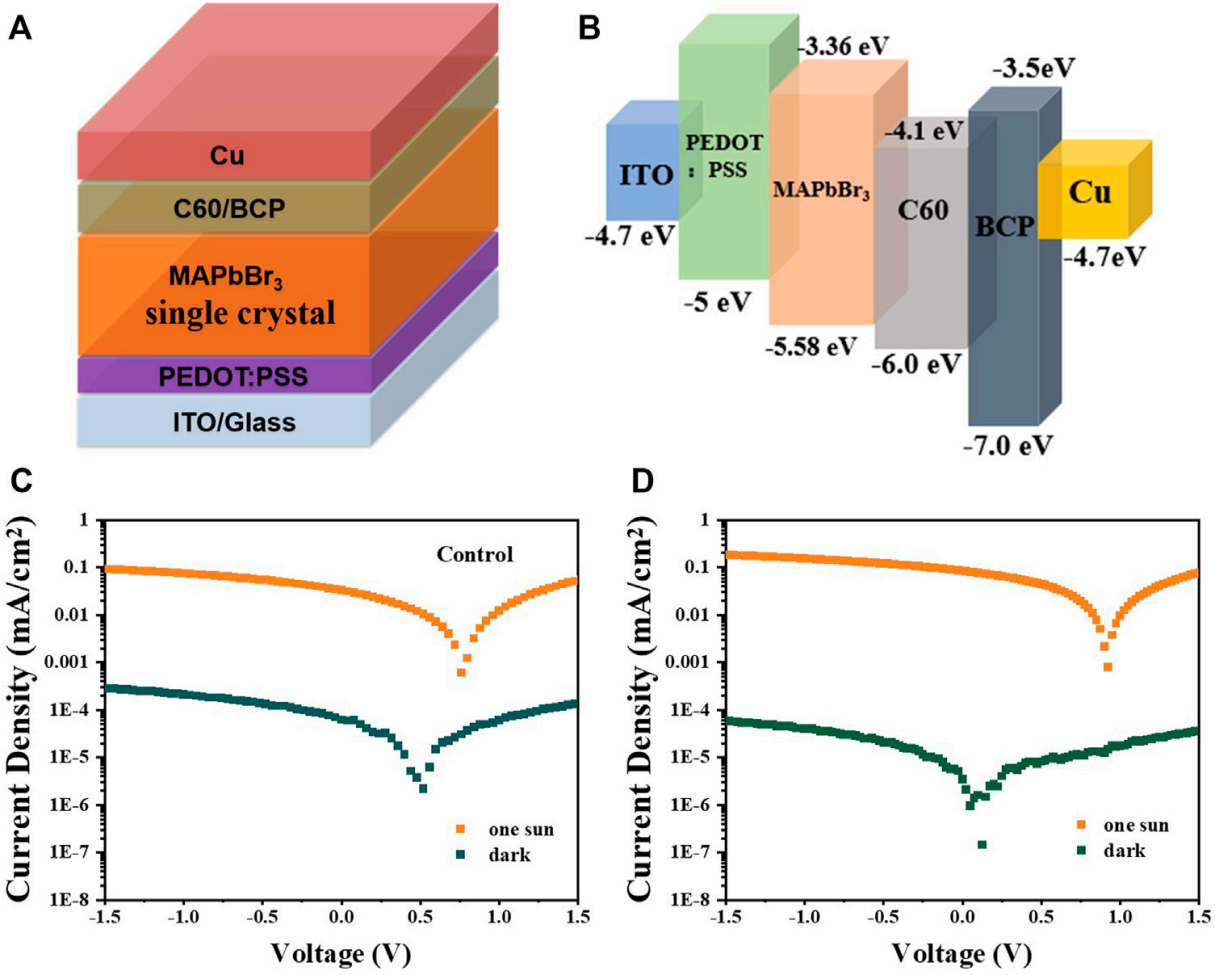
**(A)** Schematic structure and **(B)** energy band diagram of the MAPbBr_3_ single crystal–based device. Control **(C)** and O_3_–UV-treated **(D)** detectors under dark and AM 1.5 illumination.

It is obvious that O_3_ treatment enhances the optoelectronic properties of MAPbBr_3_ single-crystal wafers, which will improve the detector performance. To confirm this point, detectors with a structure of ITO/PEDOT: PSS/single crystals/C_60_/BCP/copper (Cu) are constructed (Figure 5A). The energy diagram of the device is shown in Figure 5B, which indicates that the insertion of the PEDOT: PSS hole transport layer and C_60_/BCP electron transport layer can facilitate the charge extraction. The detectors show a narrowband photo-response with a peak value in the below-bandgap absorption, similar to previously reported photodetectors based on bulk perovskite crystals. The relatively low response in the above-bandgap region is due to small penetration length and surface charge recombination. Figures 5C,D show the current density–voltage (J–V) curves of the detectors under dark and AM 1.5 illumination. Compared to the control detectors, the detectors with O_3_ treatment show a lower dark current and larger photocurrent, which is consistent with the results of lower trap density.

**FIGURE 6:**
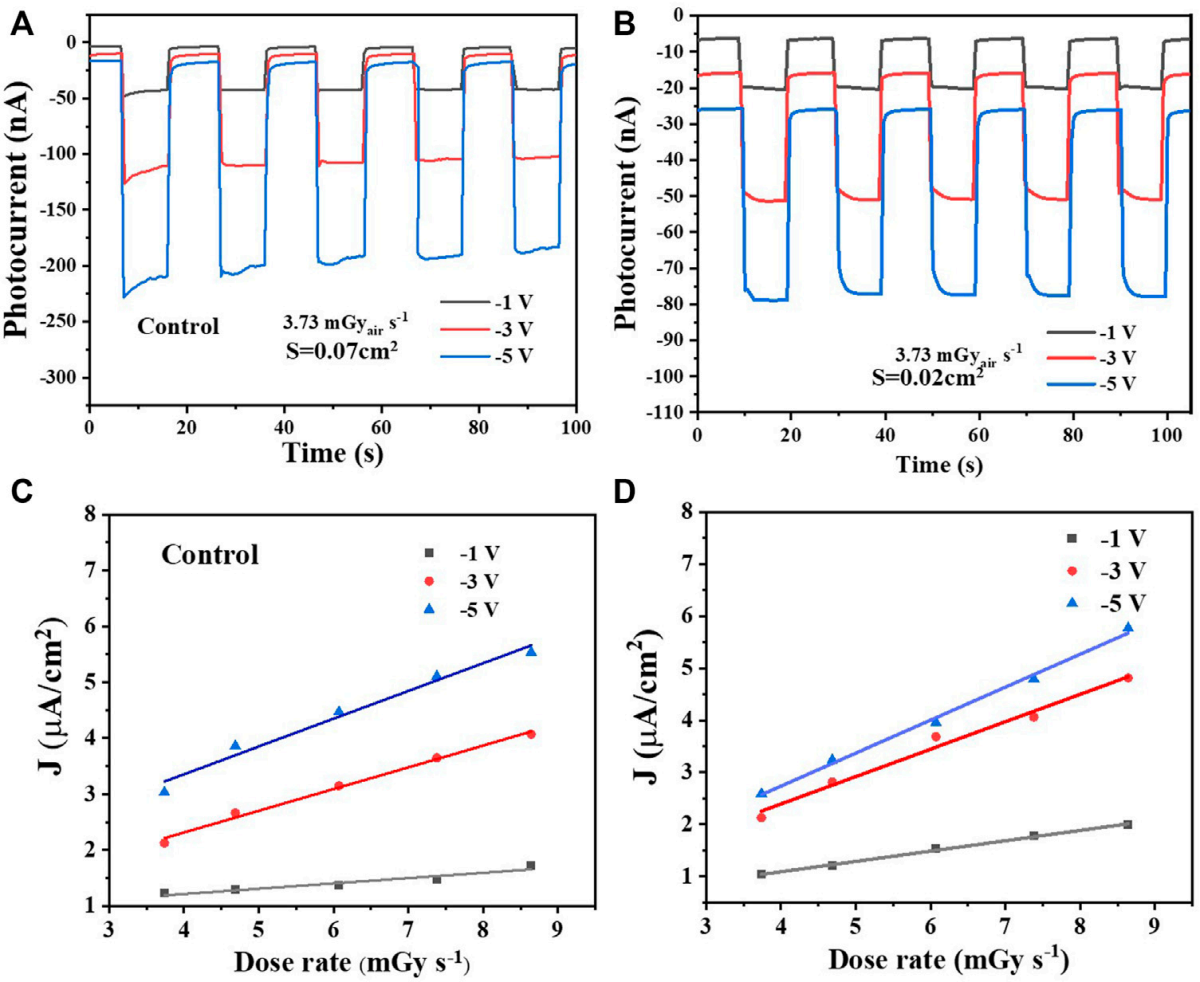
X-ray response of the control device **(A)** and O_3_–UV-treated device **(B)** under various bias. **(C)–(D)** Summary of the photocurrent of the two single-crystal detectors under various dose rates and bias.

Finally, the X-ray response properties of the detectors by continuously turning on and off the X-ray source are evaluated. The X-ray response performance of the single-crystal devices was tested under an X-ray source with energy up to 50 keV and peak intensity at 50 keV. Due to their better electrical transport properties, the X-ray response of detectors with O_3_ treatment is distinctly stronger than that of control devices under various bias **(** Figures 6A,B). Besides, it is worth noting that the dark current and photocurrent of detectors with O_3_ treatment are more stable than those of control devices, especially when the bias is increased. The smaller drift of the dark and signal current indicates ion migration is weakened after O_3_ treatment, which is consistent with the reduced trap density. Figures 6C,D summarize the photocurrent of the two single-crystal detectors under various dose rates and bias. A high sensitivity of 632 µC Gy_air_^−1^ cm^−2^ under –5 V and 525 µC Gy_air_^−1^ cm^−2^ under –1 V is obtained for the optimal devices, which surpasses that of commercial α-Se X-ray detectors.

## Conclusion

In conclusion, we develop an effective method to grow substrate-integrated, thickness-controlled, and inch-sized perovskite single crystals, which can help to reduce cross-talk between pixels and enhance vertical carrier collection. The crystal thickness can be adjusted to satisfy full attenuation of soft and hard X-ray. After polishing and O_3_ treatment, the optoelectronic properties can be improved, leading to an enhanced X-ray response. A high sensitivity of 632 µC G_air_^−1^ cm^−2^ under –5V and 525 µC Gy_air_^−1^ cm^−2^ under –1 V is obtained for the optimal devices.

## Data Availability

The original contributions presented in the study are included in the article/Supplementary Material; further inquiries can be directed to the corresponding author.
